# Sonic Hedgehog Determines Early Retinal Development and Adjusts Eyeball Architecture

**DOI:** 10.3390/ijms26020496

**Published:** 2025-01-09

**Authors:** Noriyuki Azuma, Keiko Tadokoro, Masao Yamada, Masato Nakafuku, Hiroshi Nishina

**Affiliations:** 1 Department of Ophthalmology and Laboratory for Visual Science, National Centre for Child Health and Development, Tokyo 157-8535, Japan; 2 Department of Developmental and Regenerative Biology, Medical Research Institute, Institute of Science Tokyo, Tokyo 113-8510, Japan; nishina.dbio@mri.tmd.ac.jp; 3 Department of Genetics, National Research Institute for Child Health and Development, Tokyo 157-8535, Japan; tadokoro-k@ncchd.go.jp (K.T.); yamada-m@ncchd.go.jp (M.Y.); 4 Department of Neuroscience, Graduate School of Medicine, The University of Tokyo, Tokyo 113-8654, Japan; 5 Division of Developmental Biology, Cincinnati Children’s Hospital Research Foundation, Cincinnati, OH 45229, USA

**Keywords:** Sonic hedgehog, holoprosencephaly, Pax6, fovea, retinal development, eye architecture

## Abstract

The eye primordium of vertebrates initially forms exactly at the side of the head. Later, the eyeball architecture is tuned to see ahead with better visual acuity, but its molecular basis is unknown. The position of both eyes in the face alters in patients with holoprosencephaly due to *Sonic hedgehog* (*Shh*) mutations that disturb the development of the ventral midline of the neural tube. However, patient phenotypes vary extensively, and microforms without a brain anomaly relate instead to alternation of gene expression of the Shh signaling center in the facial primordia. We identified novel missense mutations of the *Shh* gene in two patients with a dislocated fovea, where the photoreceptor cells are condensed. Functional assays showed that Shh upregulates *Patched* and *Gli* and downregulates *Pax6*, and that *Shh* mutations alter these activities. Gain of function of Shh in a chick embryo retards retinal development and eyeball growth depending on the location of Shh expression, while loss of function of Shh promotes these features. We postulate that a signaling molecule like Shh that emanates from the face controls the extent of differentiation of the neural retina in a position-specific manner and that this may result in the formation of the fovea at the correct location.

## 1. Introduction

**Figure 1:**
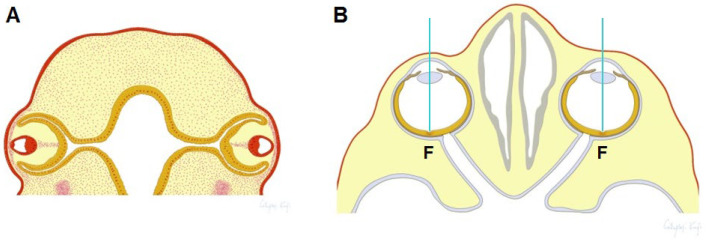
Schematic depiction showing changes in the position and architecture of the eyeball of humans during development. The architectures of the eye at an early (**A**) and later stage (**B**) of development show the establishment of horizontal asymmetry of the eyeball, the position of the fovea (F in panel **B**), and the visual axis (blue lines in panel **B**).

In most vertebrates, eyeball structures have asymmetry on the horizontal plane, as the temporal side of the eyeball is larger than the nasal side to facilitate forward vision. At early stages of development (Hamburger–Hamilton [HH] stages 8–10 of chick embryos and 4–5 weeks gestation in humans), the primordium of both eyes begins to form on each lateral side of the head. At these stages, the shape of the eyeballs and the distribution of the progenitor cells in the primitive retina are symmetrical along the anteroposterior axis of the eye, which is perpendicular to the anteroposterior axis of the whole body (Figure 1A). The production of the retinal progenitor cells first progresses from the optic nerve head to the periphery, which results in a posteroanterior gradient of cell density [1,2].

Subsequently, the eyeballs turn inward so that they are oriented forward in the same direction (HH stages 16–30 of a chick embryo and 6–12 weeks gestation in humans). In the asymmetrically shaped eye, visual cells in the retina accumulate and specialize to contribute to better vision at the posterior end point of the eye. This region is known as the fovea or area centralis. The image of an object becomes centered on this region, which is therefore the endpoint of the visual axis (Figure 1B) [1,2,3]. Concomitant with the rotation of the eyeballs at an early stage of development, the distribution of retinal progenitor cells becomes asymmetric, and they especially accumulate and differentiate at the prospective fovea or in the area centralis region. The differentiation of the retinal cells then progresses from the future fovea area to the periphery, which results in a gradient of visual sensitivity [1,2]. It is thought that retinal growth influences eyeball growth [4,5], and that the accumulation of retinal cells in the posterior temporal area may cause a larger growth in the temporal side of the eyeball than in the nasal side. However, the molecular bases of the developmental processes that lead to the gradient of retinal cell density and particular eye positions and directions are not well elucidated.

The position of the fovea/area centralis on the horizontal plane of the eyeball varies among animal species, while that on the vertical plane is mostly central. Its position on the horizontal plane does not correlate with the distance from any other eye structures, including the optic nerve head, cornea and lens. The eyeball of each animal species varies in shape, size and location in the head and orbit. Thus, it is unlikely that signaling molecules of positional markers emanate from the eye, orbit and brain tissues. In contrast, it is located on each side of the face in a manner that facilitates forward vision (Figure 1B). For example, the accumulation of retinal cells is temporally shifted in the laterally directed eyes of many fish and birds, while it is shifted posteriorly in the frontally directed eyes of the seahorse and owl [3]. These findings provide a hypothesis that a gradient of morphogens emanating from the face center determines the position of the fovea/area centralis, gradient of retinal cell density and eyeball asymmetry [6].

The position of both eyes in the face and the direction of the visual axis alter in some human anomalies. Holoprosencephaly (type 3, OMIM 142945) is a developmental defect of the forebrain and face and is caused by mutations in the *Sonic hedgehog* (*Shh*) gene [7,8]. Shh, a secreted signaling molecule, is expressed in the ventral midline tissues, such as the notochord and floor plate, and generates a ventrodorsal gradient that directs neuronal cell identity and fates in the ventral neural tube [9,10]. The phenotypes of holoprosencephaly patients vary extensively along a continuous spectrum. The most severely affected cases have only one eye and their cerebral hemispheres are fused [7]. More mildly affected cases show a variety of facial dysmorphisms with minimal anomalies in brain structure. Sometimes these patients even have a normal brain. The facial dysmorphogenesis observed in mild holoprosencephaly patients relates to the disruption of the Shh signaling center in the facial primordium [11,12,13]. Although the single eye of the severely affected cases does not contain the full architecture of the retinal tissues, no ocular anomalies have been described in the bilaterally separated eyes of patients with mild holoprosencephaly, apart from one case with coloboma [8]. We identified two patients with nonsyndromic mild holoprosencephaly associated with foveal dislocation and Shh mutations. This suggests that Shh may regulate the positioning of the fovea in the eyeballs.

Patients with foveal hypoplasia have been found to bear mutations in the *PAX6* gene [14,15], the master control gene for eye morphogenesis in both vertebrates and invertebrates [16,17,18,19]. At the early stages of development, *Pax6* is ubiquitously expressed and contributes to the growth and differentiation of retinal cells [20,21]. When the various retinal cells form asymmetrically, the regional expression patterns of *Pax6* change [22]. This suggests that the regional expression of *Pax6* is tightly linked to the asymmetric differentiation of the retinal cells. However, little is known about the regulation of regional differences of *Pax6* expression in the retina, although previous studies have explored mechanism and genes involved in differentiation of the retinal area [23,24,25].

Shh is known to antagonize Pax6 activity. In mutant zebrafish embryos that lack the Shh homologue, ectopic expression of Pax6 is observed in a bridge of tissue around the anterior pole of the neural keel that usually forms the optic stalk at a very early stage of development [26]. In the *Xenopus* optic stalk/vesicle/cup development, misexpression of BMP4, an antagonist of Shh, causes expanded expression of Pax6 at the expense of the ventral and proximal markers Vax2 and Pax2, and when Shh is misexpressed, the reverse expression is observed [27]. Increased Shh expression at the embryonic midline of blind cavefish reduces Pax6 expression, resulting in developmental arrest and degeneration of the eyes [28]. Thus, Shh plays an important role in conjunction with Pax6 in forming the correct ocular architecture at early stages of development. At middle and late stages of development, Shh is expressed in retinal ganglion cells and amacrine cells soon after differentiation (HH stage 24 of chick embryos and E14.5 of mouse embryos), albeit at very low levels, and is required for the differentiation of ganglion cells, amacrine cells, Müller glial cells and rod photoreceptors [29,30,31,32,33]. Since positioning of the foveal formation is achieved between these two stages (i.e., 8–10 weeks human gestation) [1,2] and the face primordium is the major source of Shh at these stages, we speculate that the asymmetric differentiation of the retinal cells and the rotation of the eyeballs are controlled by the Shh signals emanating from the face primordium and the *Pax6* gene in the retina. Supporting this hypothesis, we found by gain of function and loss of function experiments in chick embryos that the dose of Shh signaling is critical for both the rotation of the eyeballs and the differentiation of the retinal cells.

## 2. Results

### 2.1. Shh Mutations Detected in Patients with Foveal Dislocation

We identified two patients with nonsyndromic mild holoprosencephaly associated with foveal dislocation and Shh mutations.

**Figure 2:**
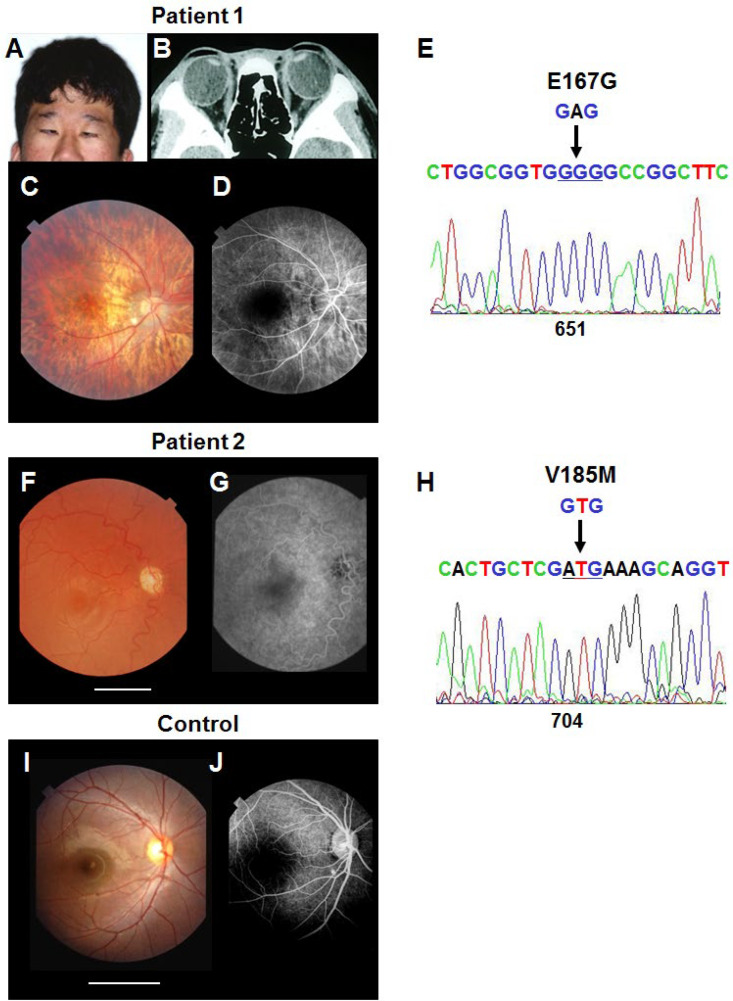
Phenotypes and genotypes of the patients carrying mutations of the *SHH* gene. (**A**–**E**): Patient 1 shows ocular hypotelorism and midface hypoplasia (**A**). CT shows enlargement of both eyeballs (more than 30 mm in axial length) (**B**). Fundus photography (**C**) and fluorescein angiography (**D**) show a small optic nerve head and foveal dislocation close to the optic nerve head. These features are present in both eyes, but only the results of the right eye are shown here. Sequencing (**E**) shows a nucleotide substitution at position 651 in exon 2 of the *SHH* gene that results in the amino acid substitution E167G. (**F**–**H**): Patient 2. Fundus photography (**F**) and fluorescein angiography (**G**) of the right eye reveal foveal hypoplasia and dislocation close to the optic nerve head. (**H**) Sequencing of the mutated and normal alleles of patient 2 shows a nucleotide substitution at position 704 in exon 2 of the *SHH* gene that results in the amino acid substitution V185M. (**I**,**J**) A photograph and fluorescein angiogram of the eye of a 10-year-old boy are presented as a normal control. The white lines indicate the distance between the fovea and the optic nerve head for comparison.

Patient 1, a 14-year-old boy, has ocular hypotelorism, midface hypoplasia, esotropia and mental retardation (Figure 2A). Computed tomography (CT) showed diffuse brain atrophy with separated cerebral hemispheres, indicating a microform of holoprosencephaly. CT also revealed enlargement of both eyeballs (Figure 2B). Ocular examinations detected a small optic nerve head and retinal degeneration in the peripheral fundus. The central fovea was dislocated close to the optic nerve head (Figure 2C,D). The patient has a normal karyotype (46XY). A sequence analysis identified a variant [c.651A>G, p.(E167G)] in the *SHH* cDNA (accession no. Q15465) (Figure 2E).

Patient 2, a 6-year-old girl, has slight ocular hypotelorism and midface hypoplasia, but CT failed to detect any brain anomaly. Her visual acuity was 0.5 and 0.6 in the right and left eye, respectively, after correction with spectacles. Eye examinations detected that the fovea was bilaterally hypoplastic and dislocated close to the optic nerve head (Figure 2F,G). She has no other abnormalities and shows normal growth, intelligence and karyotype (46XX). This patient had a variant [c.704G>A, p.(V185M)] (Figure 2H). Other gene mutations that relates to holoprosencephaly, including *ZIC2*, *SIX3*, *TGIF1*, *CDON*, *FGFR1*, *CENPF* and *DHCR7*, were not identified. Neither mutation was detected in other family members of either pedigree, all of whom were apparently normal, which indicates that these mutations are sporadic.

Since *SHH* is expressed in the retina during the middle to late stages of development, its mutations may cause retinal malformations, including the foveal hypoplasia found in patient 2. However, it is difficult to attribute the foveal dislocation observed in both patients to the dysfunction of the Shh that is expressed in the retina itself.

### 2.2. Assessment of the Functional Consequences of the SHH Mutations by In Vitro Functional Assay

**Figure 3:**
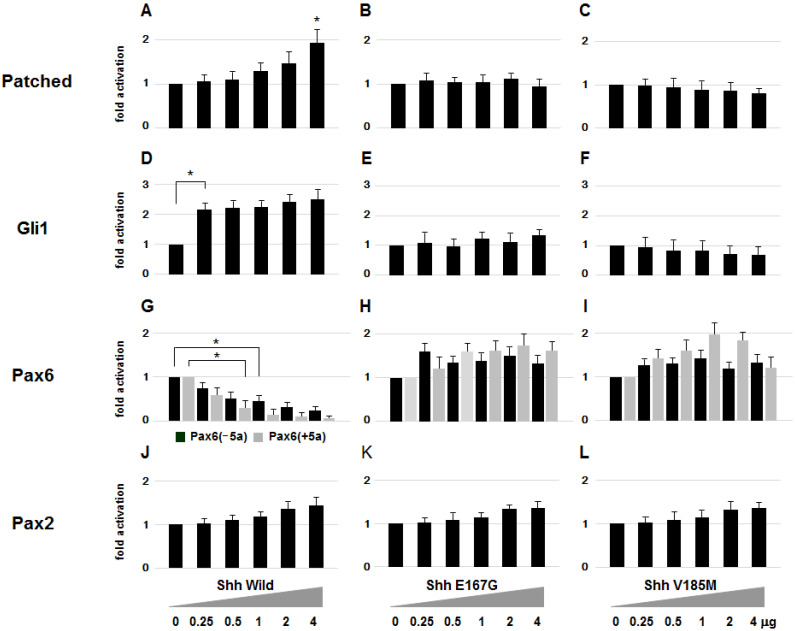
Effects of Shh and its mutants on *Patched*, *Gli*, *Pax6* and *Pax2* expression. The mRNA levels of these genes were measured in P19 cells after a *Shh* expression plasmid that generates the Shh-N recombinant protein was added. Patched (**A**) and Gli1 (**D**) expression increases with increasing amounts of the wild-type form of Shh, while Pax6 is suppressed (**G**). Pax2 also increased slightly (**J**). The E167G (**B**,**E**,**H**,**K**) and V185M (**C**,**F**,**I**,**L**) mutants disturb the increase in Patched (**B**,**C**), Gli1 (**E**,**F**) and Pax2 (**K**,**L**) and decrease in Pax6 (**H**,**I**) expression. The bar graph is shown as mean ± standard deviation (n = 3) of expression ratio of each tissue to the optic vesicle at stage 12. The photograph of qRT-PCR analysis under the bar graph is representative of three independent experiments. *, *p* < 0.05 (unpaired *t*-test, two-tailed); error bars, ±SD.

Since the *SHH* mutations in our patients are sporadic, we performed a functional assay to assess whether the detected mutations really are responsible for the phenotypic changes observed in the patients. In the Shh signaling pathway, Patched is expressed as a component of the Shh receptor complex in its target cells [29,34]. Other genes that are associated with *Shh* are the *Gli* genes, which are homologues of *Drosophila cubitus interruptus* and encode proteins with five zinc finger motifs and function as intracellular mediators of signaling [35,36]. Thus, we examined the effects of the wild-type Shh and its mutants on the expression of *Patched*, *Gli* and *Pax6*. Mouse embryonic carcinoma P19 cells, which are frequently used for the functional analysis of the *Pax6* gene, were transfected with mouse *Shh* cDNA encoding the N-terminal half of the product (*Shh-N*) [36]. After 72 h, total RNA was isolated and analyzed by qRT-PCR for the expression of the mouse *Pax6*, *Patched*, *Gli1*, *Gli2*, *Gli3*, and *Pax2* genes. Increasing amounts of *Shh* cDNA caused the expression of the endogenous *Patched* and *Gli1* genes to increase, while *Pax6* expression decreased in a dose-dependent manner (Shh Wild in Figure 3). The expression of *Pax2* also slightly increased, however, that of *Gli2* and *Gli3* was unaffected.

To analyze the effect of the Shh mutations discovered in this study, P19 cells were also transfected with the expression plasmid carrying mouse *Shh-N* cDNA into which these mutations were introduced by in vitro mutagenesis. In contrast to effects of the wild-type Shh, neither of two mutant *Shh* genes activated *Gli1* expression or suppressed *Pax6* (ShhE167G and ShhV185M in Figure 3). These results indicate that the mutations in patients are likely to cause loss of Shh function.

### 2.3. Shh Is Expressed in the Face Center at an Early Stage of Development

**Figure 4:**
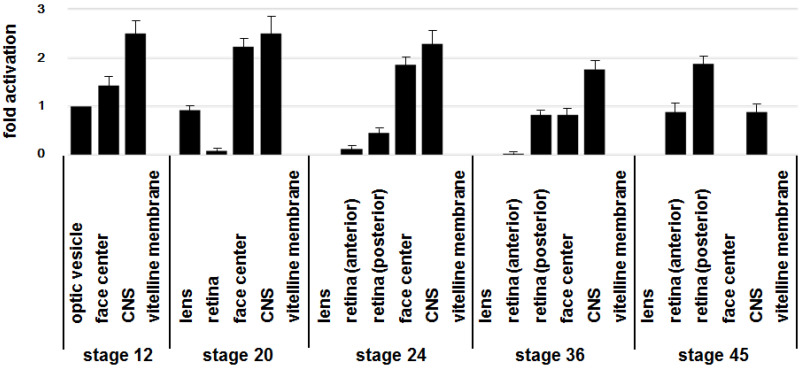
qRT-PCR analysis of the regional expression levels of *Shh* during chick embryogenesis. The indicated tissues were dissected from chick embryos at the indicated developmental stage. Relative mRNA expression was calculated using the value derived from the optic vesicle at stage 12. Vitelline membrane tissues were used as a negative control. The amount of cDNA pools used in each lane is almost constant, as demonstrated by the amplification of *β*-actin. The expression of *Shh* is more prominent in the face center and CNS at earlier stages, but at later stages dominates in the retina. The result shown is representative of three independent experiments.

To confirm the previous observations regarding regional *Shh* expression [9,11,12], we studied the expression of *Shh* during eye development using qRT-PCR. mRNA was isolated from sectioned tissues of chick embryos at HH stages 12–45. The RNA was then converted into cDNA with reverse transcriptase. The amounts of *Shh* mRNA in each tissue were analyzed by qPCR with specific primers for *Shh* (Figure 4). *Shh* was expressed in the hindbrain of the central nervous system (CNS) throughout the developmental stages examined. In the face center, its expression lasted through to stage 36 but disappeared at stage 45. Expression of *Shh* in the lens was detected at stage 20 but faded at stage 24. Contrary to this, retinal expression starts at stage 24. The levels of *Shh* expression are higher in the posterior retina than in the anterior retina. These results suggest that the face is a major source of *Shh* between stages 12 to 24, when the eyeball turns to the facial position and gains horizontal asymmetry.

### 2.4. In Ovo Misexpression of Shh Suppresses the Growth of the Retina and Eyeball

To analyze the effects of Shh on ocular development, we misexpressed Shh together with GFP in the mesenchymes around chick eyes during early developmental stages by in ovo electroporation [37]. The areas expressing the exogenous gene were revealed by the fluorescent signals of the green fluorescence protein (GFP) that was derived from GFP in pCAGGS-Shh-GPF plasmid (Figure S1A) [38]. When Shh was expressed in a relatively broad area around the eyes during HH stages 12–20, microphthalmos occurred 8 days after treatment, with thinner but normal retinal layer structures (Figure S1B–D). Cross-sections were subjected to in situ hybridization or immunohistochemistry with probes or antibodies specific for transcription factors that regulate the proliferation of retinal progenitor cells and the specification of cell fate. *Musashi*, which encodes a neural RNA-binding protein, is highly enriched in neural precursor cells [39]. *Six3*, a homologue of *Drosophila* homeobox gene *sine oculis*, is expressed early on in the optic vesicle, turns off in the future pigment epithelium, and becomes restricted to the prospective NR and to the lens placode. In the NR development, *Six3* is expressed in the entire undifferentiated neuroepithelium, then in differentiating cell layers, including the inner and outer nuclear layer and ganglion cell layer [40]. *Rx*, a paired-class homeobox gene, is expressed early on in the optic vesicle and later in the inner nuclear layer, presumably in bipolar cells of the developing NR [41]. Islet1, a homeodomain-containing transcription factor that is expressed in the ganglion cells in the developing retina [42]. The in situ hybridization and immunohistochemistry resulted in slightly weak staining of the correct layers in the affected eye compared with the unaffected eye, after quite a while (8 days) had passed (Figure S1). Expression of *Musashi* was unchanged in the treated eye.

**Figure 5:**
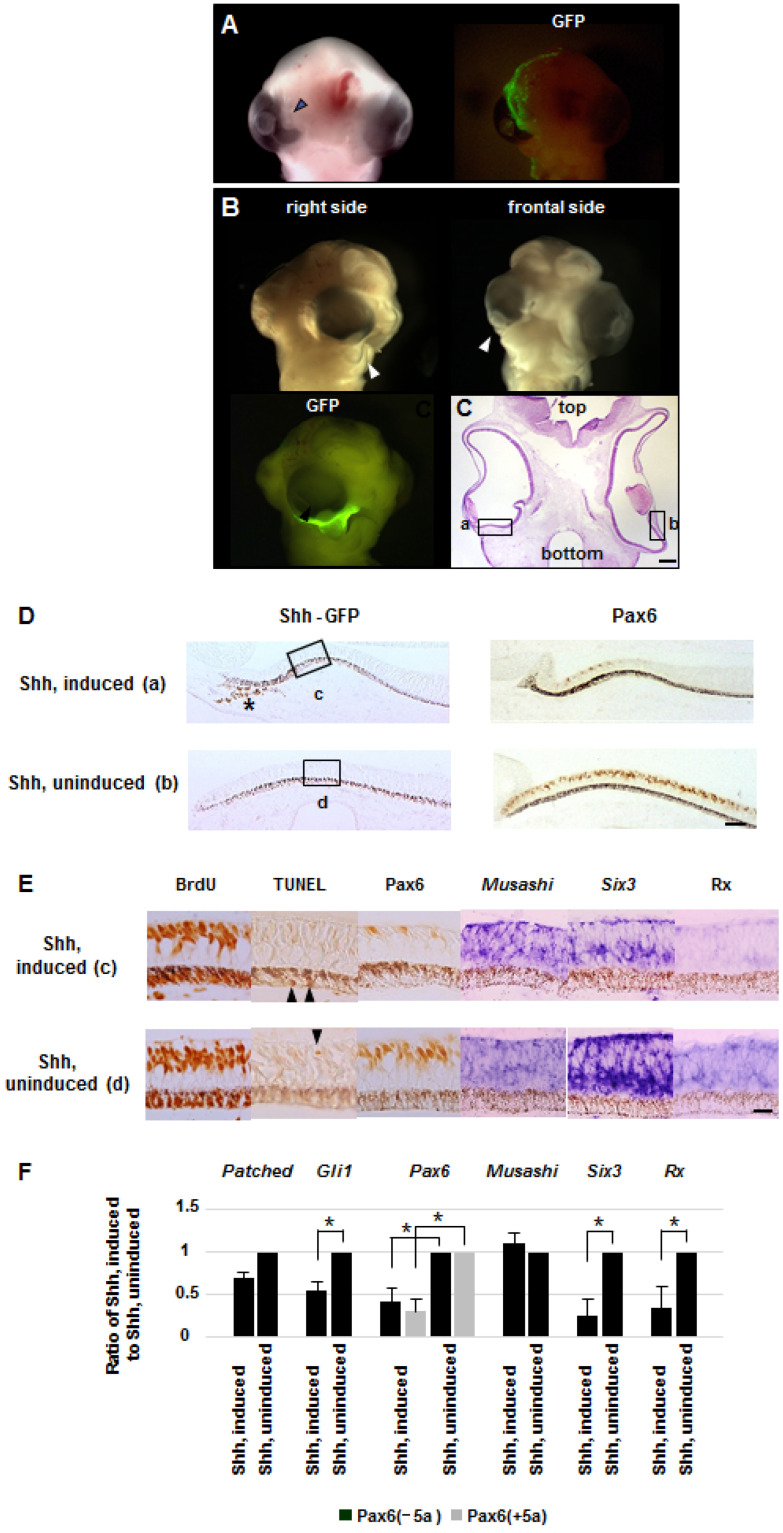
Developmental anomalies of the retina and eyeball caused by local misexpression of exogenous *Shh*. A DNA solution containing the *Shh* and *GFP* expression plasmids was electroporated in a restricted area around the right eye primordium of HH stage 14 chick embryos, and the resulting morphology was examined at stage 26. (**A**) Views of the affected eye and its fluorescent image show that the injected constructs are expressed in the upper anterior portion of the right eye. The growth of the upper anterior half of the eyeball is retarded and the cornea and lens have developed in an upward anterior direction (arrowhead). Ten times magnification by stereo microscope. (**B**) Views of the affected eye and its fluorescent image matched with the upper panel show that the injected constructs are expressed in the lower part of the right eye. The growth of the lower half of the eyeball is retarded and the cornea and lens developed in a downward direction (arrowhead). (**C**) The coronal section of the eyes shows that the growth of the bottom half of the right eyeball is retarded (inset a), and the cornea and lens are at the bottom corner (HE staining, bar scale 60 μm). (**D**) Immunohistochemistry with specific antibodies reveals exogenous Shh and GFP protein (asterisk) in the treated area of the Shh-induced right eye (inset a in panel **C**) but not in the uninduced left eye (inset b in panel **C**). There is less Pax6 expression in the treated area of the Shh-induced eye compared to the same area of the uninduced eye (bar scale 30 μm). (**E**) Immunohistochemistry with an anti-BrdU antibody shows slightly weak staining in the Shh-induced area (inset c in panel **D**) compared to the uninduced area (inset d in panel **D**). Apoptosis is slightly detected in the retina and pigment epithelia (arrowheads) in the Shh-induced and uninduced eyes, suggesting that gene induction did not result in cell death. Immunohistochemistry and in situ hybridization suggest decreased expression of *Pax6*, *Six3* and *Rx* in the Shh-induced right eye (inset a in panel **C**) compared to the uninduced left eye (inset b in panel **C**), but mostly the same level of *Musashi* expression in both eyes (bar scale 20 μm). (**F**) qRT-PCR analysis suggests that expression of *Gli1*, *Pax6*, *Six3* and *Rx* decreased in the affected right eye (dissected area of inset c in panel **D**) compared to the unaffected left eye (dissected area of inset d in panel **D**), but that there was mostly the same level of *Musashi* expression in both eyes. Expression of *Patched*, *Gli1*, *Pax6*, *Musashi*, *Six3* and *Rx* in the Shh-induced and uninduced retinas is indicated by qRT-PCR. The amount of the cDNA pools was prepared for qRT-PCR. The bar graph indicates the ratio of Shh-induced to Shh-uninduced retinas. The analysis is representative of three independent experiments. *, *p* < 0.05 (unpaired *t*-test, two-tailed); error bars, ±SD.

Next, we investigated Shh misexpression in restricted areas and immediate phenotype changes of the eye depending on the location. When Shh was expressed in the upper part of the mesenchyme surrounding the eye, the growth of the top half of the eyeball was retarded, and the cornea and lens shifted upward in 2–3 days after treatment (Figure 5A). In contrast, when Shh was expressed in a lower segment, growth of the bottom half of the eyeball was retarded, and the cornea and lens developed near the bottom (Figure 5B,C). The immunohistochemistry and in situ hybridization demonstrated that expression of *Pax6*, *Six3* and *Rx* was substantially suppressed in specific regions near the Shh expression site (Figure 5D,E). Expression of *Musashi* was unchanged in the treated eye. Low expression of *Gli1*, *Pax6*, *Six3* and *Rx* in the treated area of the retina was also identified by qRT-PCR (Figure 5F).

Although more than 90 alternative splice isoforms have been reported [43], the two isoforms with paired domain with exon 5a (*Pax6*(+*5a*)) and without exon 5a (*Pax6*(−*5a*)) play an important role for eye development. The *Pax6*(+*5a*) isoform has an additional 14 amino acid residues encoded by exon 5a in the DNA-binding domain, the paired domain (PD), and each respective isoform has different DNA-binding properties. Pax6(+5a) is expressed especially in the retinal portion where visual cells accumulate during eye development and, when overexpressed, induces a remarkable well-differentiated retina-like structure [44]. Because the anti-Pax6 antibody we used reacts with both Pax6(−5a) and Pax6(+5a), the immunohistochemistry is unable to detect changes of the isoform expression, while the qRT-PCR analysis revealed both Pax6 isoforms are suppressed (Figure 5F).

Since Pax6 promotes retinal progenitor proliferation [44], immunohistochemistry with the anti-5-bromo-2′-deoxyuridine (BrdU) antibody revealed slightly weak staining in the electroporated region where Pax6 was suppressed. Ectopic apoptosis was not detected compared with untreated areas, which suggests that high ectopic Shh signaling represses Pax6 and results in the retardation of retinal growth (Figure 5E). Electroporation of the empty vector alone, the pCAGGS-GFP or both did not induce any morphological change. The Shh-mediated growth retardation of the retina and eyeball occurred specifically at stages 12–20. The incidence of the Shh-dependent eye architectural changes at each stage is available in Supplementary Table S1.

### 2.5. In Ovo Injection of Cyclopamine Promotes the Growth of the Retina and Eyeball

**Figure 6:**
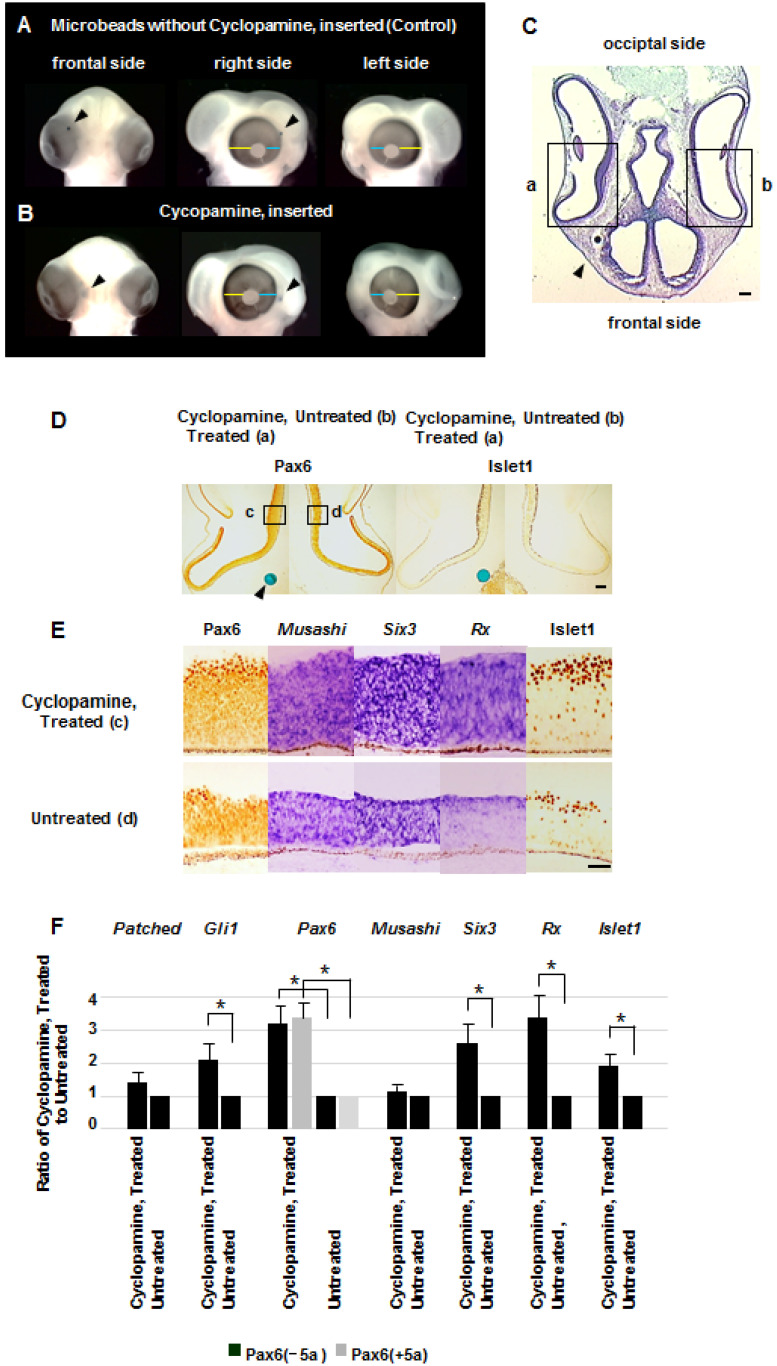
Developmental anomalies of the retina and eyeball after injecting cyclopamine. Microbeads soaked with 100 nM cyclopamine were inserted into the anterior part of mesenchymes around the right eye of stage 14 chick embryos; the resultant morphology is shown at stage 24. The anterior part of the affected eyeball near the cyclopamine beads (arrowheads) is enlarged (**B**), compared to the control eye inserted with empty beads (arrowhead) (**A**). The blue lines indicate that the length of the anterior quarter of the right, affected eyeball is longer than that of the left, unaffected eyeball, while the yellow lines indicate that the posterior quarter of the right, affected eyeball is shorter than that of the left, unaffected eyeball (**B**). Ten times magnification by stereo microscope. Ten times magnification by stereo microscope. (**C**) A horizontal section of the eyes in panel (**B**), in which a bead soaked with cyclopamine was inserted, shows that the retina near the bead (arrowhead) has thickened and that the right eyeball has enlarged (arrow) (HE staining, bar scale 60 μm). (**D**) Immunohistochemistry shows increased Islet1 expression in the periphery of the treated eye (arrow; in inset a in panel **C**), compared to the untreated eye (in inset b in panel **C**) (bar scale 30 μm). (**E**) Immunohistochemistry and in situ hybridization suggest increased expression of *Pax6*, *Six3*, *Rx* and *Islet1* in the affected right eye (inset c in panel **D**) compared with the unaffected left eye (inset d in panel **D**), but mostly the same level of *Musashi* expression in both eyes (bar scale 20 μm). (**F**) qRT-PCR analysis suggests that expression of *Gli1*, *Pax6*, *Six3*, *Rx* and *Islet1* increased in the affected right eye (dissected area of inset c in panel **D**) compared with the unaffected left eye (dissected area of inset d in panel **D**), but there is mostly same level of *Musashi* expression in both eyes. The bar graph is shown as mean ± standard deviation of the expression ratio of the treated right eye to the untreated left eye. The analysis is representative of three independent experiments. *, *p* < 0.05 (unpaired *t*-test, two-tailed); error bars, ±SD.

To analyze the effect of reducing Shh signaling on the development of the retina and eyeball, we next inserted microbeads soaked with cyclopamine, which represses Shh signaling [45], into HH stage 8–30 chick embryos. Since endogenous *Shh* is expressed in the center of the facial primordium, the beads were inserted into the anterior part of the mesenchymal tissue surrounding the eye. As the amount of cyclopamine in beads increased, the anterior half of the eyeball expanded, the rotation of the cornea and lens anteriorly was disturbed, and the eyeballs were oriented laterally or posteriorly (Figure 6A,B). The retinal areas close to the implanted beads thickened (Figure 6C,D). Immunohistochemistry and in situ hybridization suggested that expression of Pax6, *Six3*, *Rx* and Islet1 increased, but *Musashi* expression was unchanged. qRT-PCR analysis suggested that *Gli1*, *Pax6*, *Six3*, *Rx* and *Islet1* also increased. The qRT-PCR analysis revealed both Pax6 isoforms (*Pax6*(−*5a*) and *Pax6*(+*5a*)) are upregulated (Figure 6E,F). Implantation of control beads (Figure 6A) or the insertion of beads soaked with cyclopamine in other extraocular mesenchyme areas (upper, lower or posterior areas) failed to cause any anomalies. The cyclopamine-mediated growth promotion of the retina and eyeball occurred specifically at stages 12–20, when Shh was highly expressed in the face center (Figure 4). The incidence of the cyclopamine-dependent eye architectural changes at each stage is available in Supplementary Table S1. Overall, these data indicate that *Shh* expression in the frontal space around the eye retards retinal growth.

## 3. Discussion

The present study demonstrated that the Shh protein emanating from the extraocular space affects retinal organization and eyeball structure. Patients carrying missense mutations of the *Shh* gene showed a dislocated fovea and/or eyeball enlargement. Shh is expressed in the facial center for a short period, at which time the eyeball turns from a lateral position towards the face. At this stage, gain of function of Shh retards the development of the retinal layer and eyeball size and turns the eye to an abnormal position. In contrast, loss of function of Shh at the same stage promotes retinal development and eyeball growth. These experiments used various animals including human genotype–phenotype, mice cells and chick embryo, because the N-terminal half of the *Shh* gene is highly conserved among animal species [36,46]. They may be achieved by the altered expression of transcription factors that contribute to retinal formation. Gain of function of Shh induced down-regulation of Pax6, Six3 and Rx expression, and loss of function results in reverse expression patterns. Because gain of function of Pax6 promotes the growth of the retina and eyeball size at this stage [43], Shh may retard their growth via suppression of Pax6. Pax6, Six3 and Rx are expressed in the developing neural retina [20,21,40,41], and the cell autonomous activity of misexpressed Pax6 causes the ectopic expression of Rx and Six3 in the ectopic retinal formation [47]. The present findings together suggest that Pax6 lies upstream of or cooperates with Six3 and Rx under the influence of Shh signaling in early retinal formation.

The Shh signaling differentially influences expression patterns of genes. At a very early stage of the optic cup/stalk development, the territory of transcription factor expression is refined by reciprocal inhibition of Pax6 and Pax2, and by elevated Pax2 expression and suppressed Pax6 under a control of secreted Shh signaling [27,48,49]. Later, Shh that is expressed in the retina plays roles in forming the retinal architecture, but in a complex manner. Shh secreted by differentiated retinal ganglion cells promotes the progression of the ganglion cell differentiation wave front that spreads from the juncture at the optic nerve to the retinal periphery, but negatively regulates ganglion cell genesis behind the wave front [49]. In the projection of the retinal ganglion cell axons into the CNS, Shh expressed from retinal ganglion cells promotes astrocyte proliferation to allow axon guidance [33], while Shh expressed in the chiasm suppresses the number and length of neurites emerging from the ganglion cell axons [50]. The molecular mechanism that underlines the different response to Shh signaling in later stages of development is not well elucidated. The present study showed that the architecture of retinal development and eyeball growth was altered by gain of function and loss of function of Shh at early restricted stages before retinal cell differentiation. Developmental histology using monkey eyes showed that the positioning of dense retinal progenitor cells in the future fovea area begins at this early stage [2]. Shh may suppress the growth of multipotent retinal progenitor cells via the Patched–Gli1 pathway and down-regulation of Pax6.

In the Pax6 DNA-binding domains, the paired domain, two structurally distinct subdomains, the N-terminal subdomain (NTS) and C-terminal subdomain (CTS), bind respective consensus sequences [21,22]. An alternative splicing exon, exon 5a, functions as a molecular switch to select specific targets, and an insertion of an additional 14 amino acid residues encoded by exon 5a in the NTS abolishes the NTS function and enhances the transactivation activity via CTS [51,52,53]. There are functional differences in the two isoforms with or without exon 5a (Pax6(+5a) or Pax6(−5a)) in retinal development: Pax6(−5a) is expressed in the entire retina, while Pax6(+5a) is expressed especially in the retinal portion where visual cells accumulate, including the presumptive fovea area. Pax6(+5a) promotes the retinal growth and, when overexpressed, induces an excessive well-differentiated retina-like architecture, while Pax6(−5a) shows a much weaker effect. These findings indicate that Pax6(+5a) relates to a developmental cascade in the prospective fovea region, although the mechanism that regulates Pax6 alternative splicing has not yet been elucidated [44]. The present in vitro and in vivo studies showed that Shh downregulates Pax6(+5a), suggesting that the expression of Pax6(+5a) is hard to be influenced in the posterior area with dense retinal cells and the fovea, which is most distant from the Shh emanating from the center of the face.

**Figure 7:**
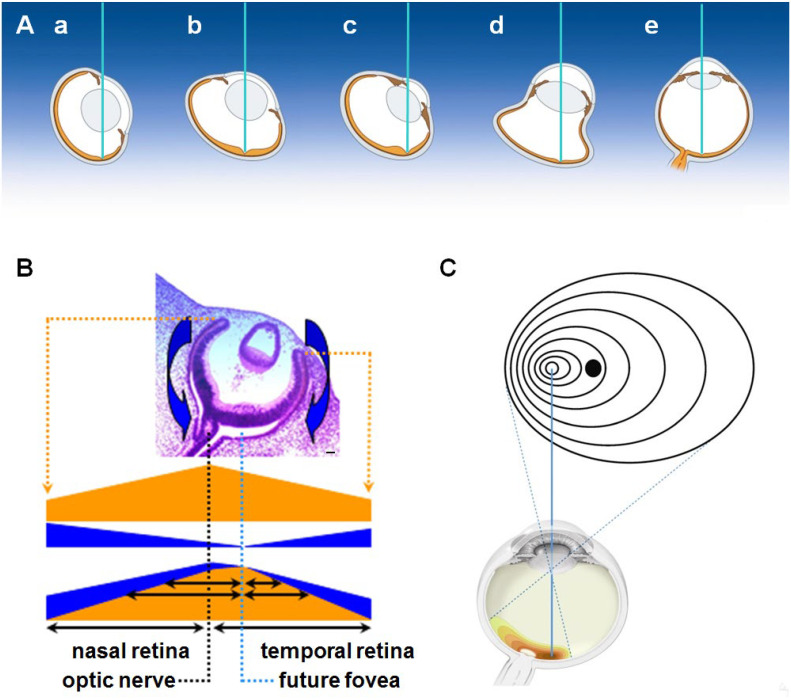
Schematic depiction showing a hypothesis to determine asymmetrical retinal growth and eye architecture, and the position of the fovea by anteroposterior gradient of suppressive factors. (**A**) The direction of the visual axis (blue lines) and position of the fovea (the posterior end of the visual axis) in various animal species. From left to right are the laterally directed eyes of many fish (a), the frontally directed eyes of the seahorse (b), the laterally directed eyes of many birds (c), the frontally directed eyes of owls (d), and the frontally directed eyes of primates, including humans (e). (**B**,**C**) Schematic depiction showing the architecture of the eyeball, retinal growth and visual field that facilitate better forward vision. (**B**) The photograph is an early developing eye at 7 week gestation. Retinal cells begins to higly proliferate in the future fovea region (bar scale 20 μm). The extent of retinal differentiation is determined by two factors. The first is a stimulating factor that is distributed along the anteroposterior gradient (in orange), and the second is a suppressing factor that emanates from the front of the face (in blue). The combination of the two factors results in the most prominent position of retinal differentiation, shifting from the posterior end to a position in the temporal area, where the fovea finally forms. (**C**) The visual field of the human eye is determined by the density of retinal cells that is similar to that in Panel (**B**). This may be a consequence of the extent of retinal differentiation, in which process Pax6 and Shh play vital roles.

A dense accumulation of retinal cells in the temporal retina, which includes the fovea, varies among animal species (Figure 7A). Its position does not correlate directly with the distance from the optic nerve head; rather, it is located on either side of the face in a manner that facilitates forward vision. For example, the accumulation of retinal cells is temporally shifted in the laterally directed eyes of many fish and birds, while it is shifted posteriorly in the frontally directed eyes of the seahorse and owl [3]. The position of the fovea subsequently determines the visual axis. The visual axis does not correlate with the anteroposterior axis of the eyeball, although the best optical pathway is through the central portion of the cornea and lens. In laterally directed eyes, the optical pathway passes through the peripheral portion of the lens and cornea, which is a poor optical pathway (Figure 7A). Since the eyeball of each animal species varies in shape, size and location in the head, it is unlikely that a common signaling center that promotes the growth and differentiation of the retinal cells and determines the position of the fovea is located in the posterior portion of the eye. Instead, it is more likely that all animal species share a graded signaling that emanates from the facial center and that serves to suppress retinal growth and to determine the position of the fovea on either side. As described below, we speculate that Shh plays a major role in this signaling gradient.

Considering these findings, we propose that the positioning of dense retinal cells with the fovea and the formation of horizontal eyeball asymmetry during ocular morphogenesis is determined by the following mechanism. At an early developmental stage, the eye primordium forms on the lateral side of the head (Figure 1A), and the density and extent of differentiation in the primitive retina are primarily determined by a posteroanterior signaling gradient of a posterior origin (which is to be the optic nerve head). This gradient proceeds as shown in orange in Figure 7B. At a later stage, a signal such as Shh that suppresses retinal development, probably by suppressing *Pax6* expression, begins to emanate from the front of the face with an anteroposterior gradient (shown in blue in Figure 7B). As a result, the position with the lowest suppressive activity shifts toward the temporal area, where retinal development becomes more prominent. Since it is thought that retinal growth influences eyeball growth [4,5], suppression of retinal growth in the nasal side and promotion of growth in the temporal side may lead to the horizontal asymmetry of the eyeball that is characterized by more growth on the temporal side of the eyeball than on the nasal side. Although we only discuss horizontal asymmetry here, it is likely that another signaling molecule exists that determines the dorsoventral pattern evident in the visual streak [54]. The visual streak is a latitudinal zone with a high density of retinal cells that is found in some flightless birds and most mammals apart from primates [1,2,3]. Thus, the signal emanating from the face modifies the fundamental anteroposterior and dorsoventral gradients and causes the fovea to finally form at the correct position in the temporal area (Figure 1B). The distance between the fovea and the temporal periphery of the retina is shorter than that between the fovea and the nasal border of the retina, and the density of the retinal cells is sharply decreased temporally but slightly decreased nasally. This organization is reflected in the elliptical visual field, which is wide temporally and narrow nasally (Figure 7C). The molecular mechanism that induces extremely high accumulation of retinal cells, including cone photoreceptors, in the fovea is not elucidated.

In most animals, including humans, the fovea is located on the temporal retina, while several bird species have a nasal fovea to facilitate lateral sight. Some bird species have two foveae in a single histologic structure, one in the temporal area and the other at the endpoint of the anteroposterior axis of the eyeball [3]. Even in this case, the positions of the two foveae can be accounted for by our model, namely, that they result from the response to two signals, one from the face and the other from anterior tissues like the lens. *Shh* expression has been detected in the developing and regenerated lens previously [55] and by present qRT-PCR analysis. It is advantageous for animals to have better visual acuity in the forward direction, as this is the direction in which they move.

The limitations of the present study included the use of chick embryo that do not have a fovea. Retinal cell development was investigated only at an early stage, but not at late maturation stages. Visual function and visual field were unable to be evaluated.

Further experiments with human retinal cell culture or organoids would support the translational relevance of the findings and clarify the downstream pathways of Shh signaling and its interactions with other developmental regulators. This newly identified implication of Shh signaling provides a new path to understand a fundamental retinal development mechanism, and has the potential to contribute to translational research, including treatment of foveal hypoplasia, to obtain better vision.

## 4. Materials and Methods

### 4.1. Ethics Statement

All analyses performed in this study were carried out in accordance with the Declaration of Helsinki and were approved by the Ethics Committee of the National Center for Child Health and Development, Tokyo, Japan (#518, genetic analysis). Written informed consent from the patients or their parents was obtained from all participants. All animal protocols were approved by the Animal Care and Use Committee of the National Research Institute for Child Health and Development, Tokyo, Japan.

### 4.2. Genetic Analysis

Genomic DNA was extracted from the peripheral blood using DNA extraction kits and guidelines (Sigma-Aldrich, St. Louis, MO, USA). Genetic analysis on holoprosencephaly-related genes, including *Shh*, *ZIC2*, *SIX3*, *TGIF1*, *CDON*, *FGFR1*, *CENPF* and *DHCR7*, was undertaken by means of Sanger sequencing in the patient and three nonaffected members of the family. The pathogenicity of the variants was determined based on the recommendation of the American College of Medical Genetics and Genomics Association Guidelines (https://www.acmg.net/).

### 4.3. Plasmid Constructs Used in the Expression Assay

The *Shh* expression plasmid used has been described previously [35,36]. Briefly, a cDNA fragment encoding the N-terminal half of mouse Shh and GFP was inserted under the control of the EF1α promoter in pEFBos. The expression plasmids bearing the mutant forms of *Shh* were generated by PCR-based in vitro mutagenesis. Sequencing analysis confirmed that the inserts bear the appropriate sequence. pCMV6-hSHH with C-terminal tGFP and pCMV6-GFP were purchased from OriGene (Rockville, MD, USA). The expression pattern of Shh was the same, but tagged GFP was weaker in the latter plasmid; thus, we used the former for Shh induction.

### 4.4. Cell Culture with Transient Transfection and qRT-PCR Analysis

Mouse embryonal carcinoma P19 cells were maintained in MEM medium (Thermo Fsher Scientific, Waltham, MA, USA) supplemented with 10% FBS at 37 °C under a humidified atmosphere of 5% CO_2_. A total of 5 × 10^4^ P19 cells were plated on 60 mm dishes, cultured for 20 h, and transfected with various amounts (0.25, 0.5, 1.0, 2.0 or 4.0 μg for each dish) of the polycationic liposome-coated expression plasmid encoding the wild-type or mutant forms of *Shh-N* (Lipofectoamine Plus, Life Technology, Carlsbad, CA, USA). An amount of 0.05 μg of pSVβgal (Promega, Tokyo, Japan) was also added as an internal control. Cell extracts were prepared after 24, 48 and 72 h.

Total RNA was extracted from the cells in each dish using RNeasy Mini Kit (QIAGEN, Tokyo, Japan) and its concentration determined using a NanoDrop LITE (Thermo Fisher Scientific, Waltham, MA, USA). Expression of mRNAs in each RNA sample was determined by two-step qRT-PCR using the StepOnePlus Real-Time PCR System (Applied Biosystems, Waltham, MA, USA). C_T_ values were normalized to the expression levels of *HPRT1* for mRNA and *7SK* for snRNA, and relative amounts of target RNAs were calculated using the 2^−ΔΔCT^ method. Primers: mouse *Pax6*, 5′-CACAGCGGAGTGAATCAGCTTG and 5′-CCAGAATTTTACTCACACAACCGT (this yields a 160 bp band for *Pax6*(−*5a*) and a 202 bp band for *Pax6*(+*5a*)); mouse *Patched*, 5′-CAGTCGTGTGCACGTCTACATGT and 5′-GCCAACTTCGGCTTTATTCAGCAT (300 bp); mouse *Gli1*, 5′-GGCTGGATCAGCTGCATCAGCT and 5′-GAAGCATATCTGGCACGGAGCAT (280 bp); mouse *Gli2*, 5′-CACTCCGCAAGCATGTGAAGACT and 5′-CGGCATCTCCATGCCACTGTCA (323 bp); mouse *Gli3*, 5′-CAGTTGCAGAGTGGCATCTCTGA and 5′-CTGGGCCATTGTAGGTACTGCTA (355 bp); mouse *Pax2*, 5′-AACAGGATCATCCGGACCAAAGTT and 5′-TCCTCGCGTTTCCTCTTCTCACC (209 bp); mouse *β-actin*, 5′-GTGGGCCGCCCTAGGCACCA and 5′-CTCTTTGATGTCACGCACGATTTC (540 bp).

### 4.5. Detection of Shh Expression by RNA Isolation and Detection

Tissues from the eye, face and floor plate of the CNS of HH stage 12–45 chick embryos were excised. Total RNA was isolated from each tissue and converted into cDNA as cited above. Expression of mRNAs in each RNA sample was determined by two-step qRT-PCR as cited above. Primers: chicken *Shh*, 5′-ACCCCGTTAGCCTATAAGCAGTT and 5′-CACCAGCTTGGTGCCTCCATG (product size, 527 bp); chicken *β-actin*: 5′-AACACAGTGCTGTCTGGTGGTAC and 5′-CAGACAGAGTACCTGCGCTCAG (134 bp).

### 4.6. In Ovo Electroporation

Fertilized eggs were purchased from Nisseizai (Tokyo, Japan). A small window was opened for access and phosphate-buffered saline was poured over the embryo to obtain appropriate resistance. A DNA solution containing the *Shh* expression plasmid and pCAGGS-GFP was introduced with a sharp glass pipette into the mesenchymes around the eye of chick embryos at HH stages 8–30. Eggs, in which early changes are examined, were also injected with 5-bromo-2′-deoxyuridine (BrdU; 0.3 mg/mL). The head of the embryo was then placed between platinum electrodes (CUY21, BEX Co., Tokyo, Japan), and electric pulses were applied (25–40 V, 90 ms, 1–6 times). The eggshells were sealed with plastic tape and the embryos were allowed to develop in a humidified incubator at 38.5 °C.

### 4.7. In Ovo Injection of Cyclopamine

Cyclopamine was purchased from CosmoBio (Tokyo, Japan). A small window was opened in the fertilized eggs and phosphate-buffered saline was poured over the embryo to preserve humidity. Analytical grade resin (Bio-Rad, Hercules, CA, USA) soaked with cyclopamine (10–100 nM, LKT Laboratories, St Paul, MN, USA) was inserted into the mesenchymes around the eyes of chick embryos at HH stages 8–30. The egg shells were sealed and the embryos were allowed to develop in humidified incubators.

### 4.8. Immunohistochemistry, Detection of Apoptosis and In Situ Hybridization

Tissues from the chick embryos were fixed in 4% paraformaldehyde. Cryo-sections (6 μm) were stained with antibodies and visualized as described previously [56]. A monoclonal antibody against mouse Shh was provided by Dr. MacMahon, and polyclonal antibodies against mouse and human Shh were purchased from the Developmental Studies Hybridoma Bank at the University of Iowa and from Santa Cruz Biotechnology, respectively. A monoclonal antibody against chicken Pax6 was a gift from Dr. Fujisawa [23]. Monoclonal antibodies that detect BrdU and GFP were purchased from DAKO (Carpinteria, CA, USA), while the antibody specific for Islet1 protein was purchased from DSHB (Iowa, IA, USA). Apoptotic cells were also determined by the TUNEL in situ labeling method using an Apoptag Kit (Intergen, Edinburgh, UK). Section in situ hybridization was performed as described [44]. Probes were prepared from plasmids that contained chick *Musashi* (EcoRI, T7 polymerase), *Six3* (HindIII, T3) and *Rx* (HindIII, T3).

### 4.9. Detection of Expression of Patched, Gli1, Pax6, Musashi, Six3, Rx and Islet1 by RNA Isolation and Detection

Tissues from the anterior part of the retina, around which Shh was misexpressed or beads soaked with cyclopamine were inserted, were excised. Total RNA was isolated from each tissue and converted into cDNA as cited above. Expression of mRNAs in each RNA sample was determined by two-step qRT-PCR as cited above.

Primers: chicken *Patched*, 5′-CAGTCGTGTGCACGTCTACATGT and 5′-GCCAACTTCGGCTTTATTCAGCAT (300 bp); chicken *Gli1*, 5′-GGCTGGATCAGCTGCATCAGCT and 5′-GAAGCATATCTGGCACGGAGCAT (280 bp); chicken *Pax6*, 5′-CGGCAGAAGATCGTGGAACTCG and 5′-GCACTCTCGTTTATACTGCGCTAT (this yields a 207 bp band for *Pax6*(−*5a*) and a 249 bp band for *Pax6*(+*5a*)); chicken *Musashi*, 5′-CCTGATTCAGTGTCATCCTGCT and 5′-GAGCTCTGCAGAGGACAACTCCT (321 bp); chicken *Six3*, 5′-AGAGTTGTCAATGTTTCAGCTGCC and 5′-TCTCGGCCTCCTGGTAGTGCG (312 bp); chicken *Rx1*, 5′-TGGAGGCCAGCTCCATGAAGCT and 5′-CGACGTCCTCCAGCGGGTACT (312 bp); chicken *Islet1*, 5′-ACCCTGGAAAGTACTGAGTGACTT and 5′-AGCGGTTCCTTGCCAGGTTCATT (330 bp).

### 4.10. Statistics

Statistical analysis was performed using Microsoft Excel. All values are expressed as mean ± S.D. Unpaired two-tailed *t*-tests were used to compare groups. *p* values less than 0.05 were considered statistically significant; individual *p* values are indicated by asterisks in graphs (*, *p* < 0.05).

## Data Availability

The data that support the findings of this study are available from the National Center for Child Health and Development but restrictions apply to the availability of these data, which were used under license for the current study, and so are not publicly available. Data are, however, available from the authors upon reasonable request and with the permission of the National Center for Child Health and Development. The corresponding author, N.A., should be contacted if anyone wants to request the data from this study.

## Supplementary Materials

The following supporting information can be downloaded at: https://www.mdpi.com/article/10.3390/ijms26020496/s1.
