# Supplementary material for: Sonic Hedgehog Determines Early Retinal Development and Adjusts Eyeball Architecture
Source: Int J Mol Sci. 2025 Jan 9;26(2):496. doi: 10.3390/ijms26020496 (PMC11764597; doi:10.3390/ijms26020496)
Supplement: Supplementary file 1 [file ijms-26-00496-s001.zip › ijms-3355113-supplementary.pdf]

Supplementary Materials

This file includes:

Supplementary Figure S1

Supplementary Table S1

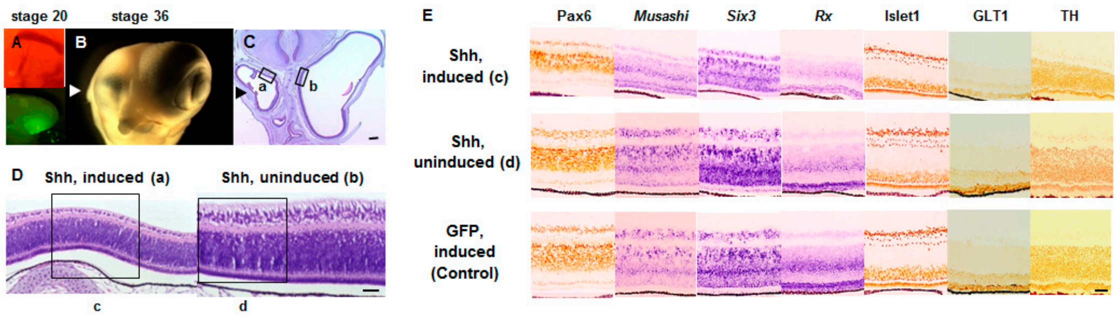

Figure S1. Developmental anomalies of the retina and eyeball caused by wide misexpressing exogenous *Shh*. A DNA solution containing the *Shh* and *GFP* expression plasmids was electroporated into the mesenchymes around the right eye primordium of HH stage 14 chick embryos. (A) Fluorescence of GFP at stage 20 indicates the transfer of the *Shh* gene in a relatively broad area around the eye. The resulting morphology examined 8 days after treatment (HH stage 36) by stereoscopic (B, 10 times magunification) and light microscopy (C) shows development of microphthalmos in the Shh-induced right eye (arrowheads), while the uninduced left eye developed normally (bar scale 60 µm). (D) Sections stained with hematoxylin and eosin [HE] show that the retinal layer in the affected right eye (inset a in panel C) is thinner than the apparently normal layer of the unaffected left eye (inset b in panel C) (bar scale 30 µm). (E) *In situ* hybridization (*Six3* and *Rx*) and immunohistochemistry (Pax6 and Islet1) suggest slightly weak staining at correct layers in the affected right eye (inset c in panel D) compared with the left eye (inset d in panel D). but mostly same level of *Musashi*, GLT1 and TH expression in both eyes (bar scale 20 µm).

Table S1 Incidence of morphological changes of the treated eyes at each stage of treatment

Experimental methods and resultant morphology	HH stage of treatment [a]						
	8	12	16	18	20	24	30
**Electroporation with the *Shh* gene in a restricted area around the eye [b]**							
Microphthalmos [c]	21[d]/43[e] [49] [f]	0/42 [0]	0/43 [0]	0/56 [0]	0/47 [0]	0/46 [0]	0/48 [0]
Retardation of regional eyeball growth	0/43 [0]	7/42 [17]	11/43 [25]	23/56 [41]	18/47 [38]	7/46 [15]	0/48 [0]
Retardation of regional retinal growth	0/43 [0]	5/42 [12]	8/43 [19]	17/56 [30]	14/47 [30]	4/46 [9]	0/48 [0]
**Insertion of microbeads soaked with cyclopamine into the anterior area around the eye [g]**							
Promotion of regional eyeball growth	0/32 [0]	14/31 [45]	14/26 [54]	18/34 [53]	13/29 [45]	2/37 [5]	0/36 [0]
Promotion of regional retinal growth	0/32 [0]	11/31 [35]	10/26 [38]	13/34 [38]	9/29 [31]	0/37 [0]	0/36 [0]

a. The eyes were sampled 3-4 days after treatment.
b. Any of morphological changes was not identified, when GFP was induced.
c. Significant microphthalmos may occur if there is a reduction of optic vesicle growth at a very early stage.
d. The number of eyes in which morphological changes were identified histologically.
e. The number of treated eyes that survive at the sampling stage.
f. Percent of morphologically altered eyes relative to the total number of surviving eyes.
g. Any of morphological changes was not identified, when microbeads only without cyclopamine were inserted.
